# Effects of Ionic Liquid Alkyl Chain Length on Denaturation of Myoglobin by Anionic, Cationic, and Zwitterionic Detergents

**DOI:** 10.3390/biom9070264

**Published:** 2019-07-08

**Authors:** Joshua Y. Lee, Katherine M. Selfridge, Eric M. Kohn, Timothy D. Vaden, Gregory A. Caputo

**Affiliations:** 1 Department of Chemistry and Biochemistry, Rowan University, Glassboro, NJ 08028, USA; 2 Bantivoglio Honors College, Rowan University, Glassboro, NJ 08028, USA; 3 Department of Molecular and Cellular Biosciences, Rowan University, Glassboro, NJ 08028 USA

**Keywords:** room temperature ionic liquids, fluorescence, protein folding, ionic liquids, heme, myoglobin

## Abstract

The unique electrochemical properties of ionic liquids (ILs) have motivated their use as solvents for organic synthesis and green energy applications. More recently, their potential in pharmaceutical chemistry has prompted investigation into their effects on biomolecules. There is evidence that some ILs can destabilize proteins via a detergent-like manner; however, the mechanism still remains unknown. Our hypothesis is that if ILs are denaturing proteins via a detergent-like mechanism, detergent-mediated protein unfolding should be enhanced in the presence of ILs. The properties of myoglobin was examined in the presence of a zwitterionic (*N*,*N*-dimethyl-*N*-dodecylglycine betaine (Empigen BB^®^, EBB)), cationic (tetradecyltrimethylammonium bromide (TTAB)), and anionic (sodium dodecyl sulfate (SDS)) detergent as well as ILs based on alkylated imidazolium chlorides. Protein structure was measured through a combination of absorbance, fluorescence, and circular dichroism (CD) spectroscopy: absorbance and CD were used to monitor heme complexation to myoglobin, and tryptophan fluorescence quenching was used as an indicator for heme dissociation. Notably, the detergents tested did not fully denature the protein but instead resulted in loss of the heme group. At low IL concentrations, heme dissociation remained a traditional, cooperative process; at high concentrations, ILs with increased detergent-like character exhibited a more complex pattern, which is most likely attributable to micellization of the ionic liquids or direct denaturation or heme dissociation induced by the ILs. These trends were consistent across all species of detergents. 1,6-diphenyl-1,3,5-hexatriene (DPH) fluorescence was further used to characterize micelle formation in aqueous solutions containing detergent and ionic liquid. The dissociation thermodynamics show that EBB- and TTAB-induced dissociation of heme is not significantly impacted by room temperature ionic liquids (RTILs), whereas SDS-induced dissociation is more dramatically impacted by all RTILs examined. Together, these results indicate a complex interaction of detergents, likely based on headgroup charge, and the active component of RTILs to influence heme dissociation and potentially protein denaturation.

## 1. Introduction

Room temperature ionic liquids (RTILs) and their interactions have been an intense area of study for the past ~20 years. Much of this work has been focused on the electrochemical and physical properties of these molecules. The material properties of these molecules are advantageous for many applications because of the variability in cation/anion pairs, electrochemical properties, negligible vapor pressure, and miscibility with many solvents [1]. Notably, it is the ability to interact with both organic and aqueous phases that has sparked the interest in the biocompatibility of RTILs. Numerous groups have recently begun investigating how ILs can be incorporated into catalytic processes, both traditional and enzymatic [2,3,4,5]. Additionally, ILs have been proposed as a good candidate for pharmaceutical and therapeutic formulations to assist in overcoming solubility barriers for some drugs [6,7,8]. There is also great interest in how RTILs may be used as additives for analytical separations of biomolecules [9]. However, more work needs to be done to characterize the fundamental types of interactions between ILs and biomolecules and their functional effects in order to develop predictive models.

In the characterization of how RTILs interact with biomolecules, there is a nearly infinite amount of complexity in the design of studies. There are unanswered questions at both the basic, fundamental levels up through the increased complexity of in vivo and ecological investigations. A number of literature reports in recent years have focused on characterizing the specific interactions of RTILs with various classes of biomolecules or individual molecules including proteins, nucleic acids, polysaccharides, and lipids [7,10,11,12,13]. These studies often target the mechanism of interaction between a subset of RTILs with a specific target [10,14,15]. Previous work from our lab has shown the functional impact of incorporating RTILs with both proteins and lipids [10,11,12,14].

Proteins are a common and reasonable system to investigate how RTILs interact with biomolecules. Proteins perform a vast array of functions in the cell, are frequently incorporated in industrial processes, and many are only marginally thermodynamically stable in the folded conformation under conditions where functionality is preserved. Numerous enzymes have been studied with RTILs such as cellulases [16], proteases [17,18], and nucleases [19]. Additionally, studies on non-enzymatic proteins have paralleled the enzyme studies with more of a focus on structural destabilization [20]. These studies have shown that RTILs can both stabilize and destabilize proteins, depending on the protein in question, molecular composition of the RTIL, concentration of the RTIL, and sample conditions. 

Myoglobin is a small, monomeric, heme-containing protein involved in oxygen storage in mammalian muscle tissue. The heme incorporated in the protein structure yields an intense absorption band at ~409 nm that disappears upon protein denaturation [12]. As such, myoglobin has been a widely characterized model to study protein folding and unfolding thermodynamics. These studies have used a wide range of denaturants, conditions, and supplements including RTILs [10]. Previous work from our groups demonstrated that RTIL molecular composition had significantly different impacts on the stability of myoglobin, as well as on the kinetics of myoglobin denaturation [10,21]. The identity of the denaturing agent, and thus the mechanism of denaturation, also impacts the influence of RTILs on the denaturation process. Experiments using the zwitterionic detergent *N,N*-dimethyl-*N*-dodecylglycine betaine showed that the inclusion of a series of RTILs had no impact on the partial denaturation and heme-loss from myoglobin. This result was intriguing since some of the same RTILs were shown previously to enhance myoglobin denaturation by guanidinium HCl (GuHCl) and urea [10,12,21]. These differences in behavior highlight the still unresolved questions of the chemical mechanism by which different RTILs stabilize or destabilize protein structure and activity.

Generally, detergent mediated protein denaturation is driven by partitioning of the hydrophobic alkyl chains of the detergent molecules into the hydrophobic core of a folded globular protein. This reduces the enthalpic cost of exposing hydrophobic residues to the aqueous milieu, as they are effectively shielded by the detergent molecules. Notably, several groups have reported on the detergent-like denaturation mechanism of RTILs [22,23]. This is driven by the presence of variable length alkyl chains attached to many common imidazolium-containing RTILs. 

**Figure 1:**
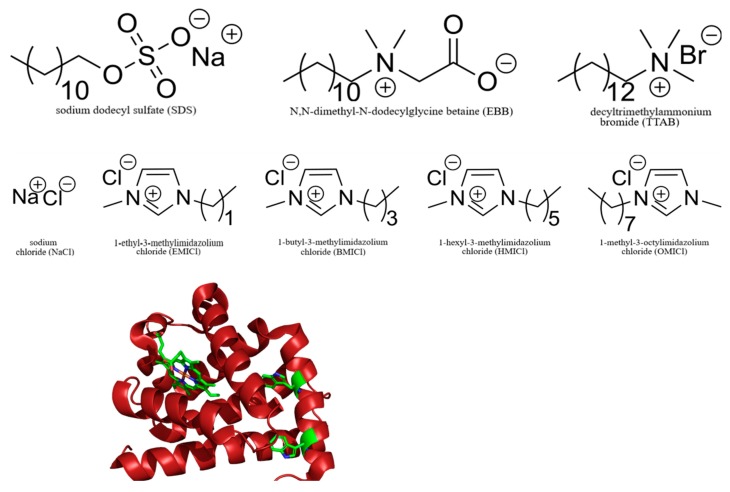
Structures of molecules used in this study. Detergents sodium dodecyl sulfate (SDS), Empigen BB^®^ (EBB), and tetradecyltrimethylammonium bromide (TTAB) are in the top row. Room temperature ionic liquids (RTILs) and the control NaCl are in the middle row. The three-dimensional (3D) structure of myoglobin from PDBID:5ZZE is shown at the bottom. In the myoglobin structure, the two Trp residues and the heme group are colored based on elemental composition, with green representing carbon.

Extending the initial work on myoglobin denaturation with detergents, the role of both detergent structure and RTIL alkyl chain length were examined in this study. A panel of RTILs which varied only in the length of the alkyl chain attached to the imidazolium were introduced at varying concentrations in the presence of either a zwitterionic (*N,N*-dimethyl-*N*-dodecylglycine betaine, EBB), anionic (sodium dodecyl sulfate, SDS), or cationic (tetradecyltrimethylammonium bromide, TTAB) detergent (Figure 1). Notably, the octylimidazolium RTIL has the alkyl chain attached to the 3-position of the imidazolium, while the other RTILs have the alkyl chain attached to the 1-position (Figure 1). The effects of these RTILs on myoglobin denaturation were investigated using a combination of absorbance, fluorescence, and circular dichroism spectroscopies, primarily focused on the spectroscopic signature of the myoglobin heme group.

## 2. Materials and Methods 

### 2.1. Materials

Horse skeletal myoglobin (95–100% lyophilized powder) and Empigen BB (Empigen BB ~30% pure liquid) were purchased from Sigma Life Sciences (St. Louis, MO, USA); myoglobin was stored at −20 °C in the dark. The RTILs: EMICl, BMICl, HMICl, OMICl, and 1,6-diphenyl-1,3,5-hexatriene (DPH) were purchased from Sigma Chemical Company (St. Louis, MO, USA). Ionic liquids were >97% purity. NaCl was purchased from Fisher Scientific (Fair Lawn, NJ, USA).

### 2.2. Sample Preparation

All samples were prepared in 2 mM sodium phosphate buffer (pH 6.9–7.2) using aqueous solutions of myoglobin, ILs, and detergent in ultrapure water. Final myoglobin concentrations were held at 0.20 mg/mL for all experiments, except low-UV circular dichroism (CD) spectroscopy which used 0.0375 mg/mL (by a factor of four). Each sample contained one species of RTIL or control salt at a concentration of 0, 50, 150, or 300 mM. Samples were created by mixing appropriate volumes of buffer, myoglobin, RTIL, and detergent with the final volume of each sample to 1000 μL. Detergents were added from concentrated stock solutions of EBB (20 mM stock), SDS (10 mM stock), or TTAB (25 mM stock). All experiments were performed at least in triplicate. A complete listing of samples examined is shown in Supplementary Table S1.

### 2.3. Absorbance Measurements

Myoglobin, ILs, and EBB were combined in buffer and allowed to incubate for 30 min at room temperature. Absorbance spectra for unfolding experiments were measured from 375–575 nm using a Thermo Scientific Genesys 10S UV-Vis Spectrophotometer (Waltham, MA, USA).

### 2.4. Fluorescence Spectroscopy

Tryptophan fluorescence dequenching was measured using a SpectraMax M5 or i3x plate reader (Molecular Devices) in clear, untreated UV-Star 96-well plates (Greiner Bio One - 655801, Kremsmünster, Upper Austria). The excitation and emission were at 280 nm and 350 nm, respectively. The RTILs used in this study presented no inner filter effects at these wavelengths. All spectra were background corrected by subtracting spectra from analogous samples lacking protein before further analysis.

### 2.5. Circular Dichroism Spectroscopy

Circular dichroism spectra were recorded on a J-810 spectropolarimeter (Jasco, Easton, MD, USA) for each concentration and species of ionic liquid, at all concentrations of detergent. Spectra were collected from 375–500 nm to monitor heme dissociation or from 190–260 nm to monitor myoglobin structure denaturation. All spectra were background corrected by subtracting spectra from analogous samples lacking protein before further analysis.

### 2.6. Calculation of Gibbs Free Energy of Dissociation

The change in Gibbs free energy of dissociation (∆G_dissociation_) in each ionic liquid environment was calculated by

(1) $$f\;=\frac{exp\;\left[-m\frac{\left(C_{m}-C\right)}{RT}\right]}{1\;+\;exp\;\left[-m\frac{\left(C_{m}-C\right)}{RT}\right]}\;,$$

where R is the ideal gas constant (8.314 J·mol^−1^·K^-1^), T is the temperature in kelvin (295 K was estimated for all experiments). In Equation (1), *f* is the fraction of unfolded myoglobin molecules. The equation is used to fit the data to two fit parameters, *m* and *C_m_*. *m* represents ∆G_dissociation_ and *C_m_* represents the concentration where half the present myoglobin is denatured. OriginLab’s Origin Pro 2019b (Northampton, MA, USA) was utilized to apply the fitting equation to the data. Statistical analysis was performed by a one way ANOVA using the JMP software suite (SAS Institute Inc., Cary, NC, USA). The remaining data visualization was aided by Daniel’s XL Toolbox add-in for Excel, version 7.3.2, by Daniel Kraus, Würzburg, Germany (www.xltoolbox.net). Sample fitting data are shown in Supplementary Figure S5.

## 3. Results

### 3.1. Critical Micelle Concentration (CMC) Analysis

In order to accurately compare the combinatorial effects of detergents on the dissociation of heme from myoglobin, any interactions between detergent and RTILs should first be characterized. Previous reports have shown that some RTILs have an inherent propensity to micellize [24], while other reports show that ionic strength can impact the critical micelle concentration (CMC) [25,26]. Previously, we showed that a different panel of RTILs did not affect the CMC of EBB [12]; however, the longer alkyl chains of the ILs in this study coupled with the ionic character of the headgroups of TTAB and SDS require this to be investigated again. The results indicate that aggregation transitions of the detergents used in this study are not dramatically impacted by the presence of 300 mM RTIL (Supplementary Figure S1). We also examined the propensity of the RTILs to micellize on their own, and showed that even at the highest concentration used in this study (300 mM), the ILs only begin to show evidence of aggregation, indicating the concentrations tested are still well below the CMCs (Supplementary Figure S1).

### 3.2. Absorbance Spectroscopy

**Figure 2:**
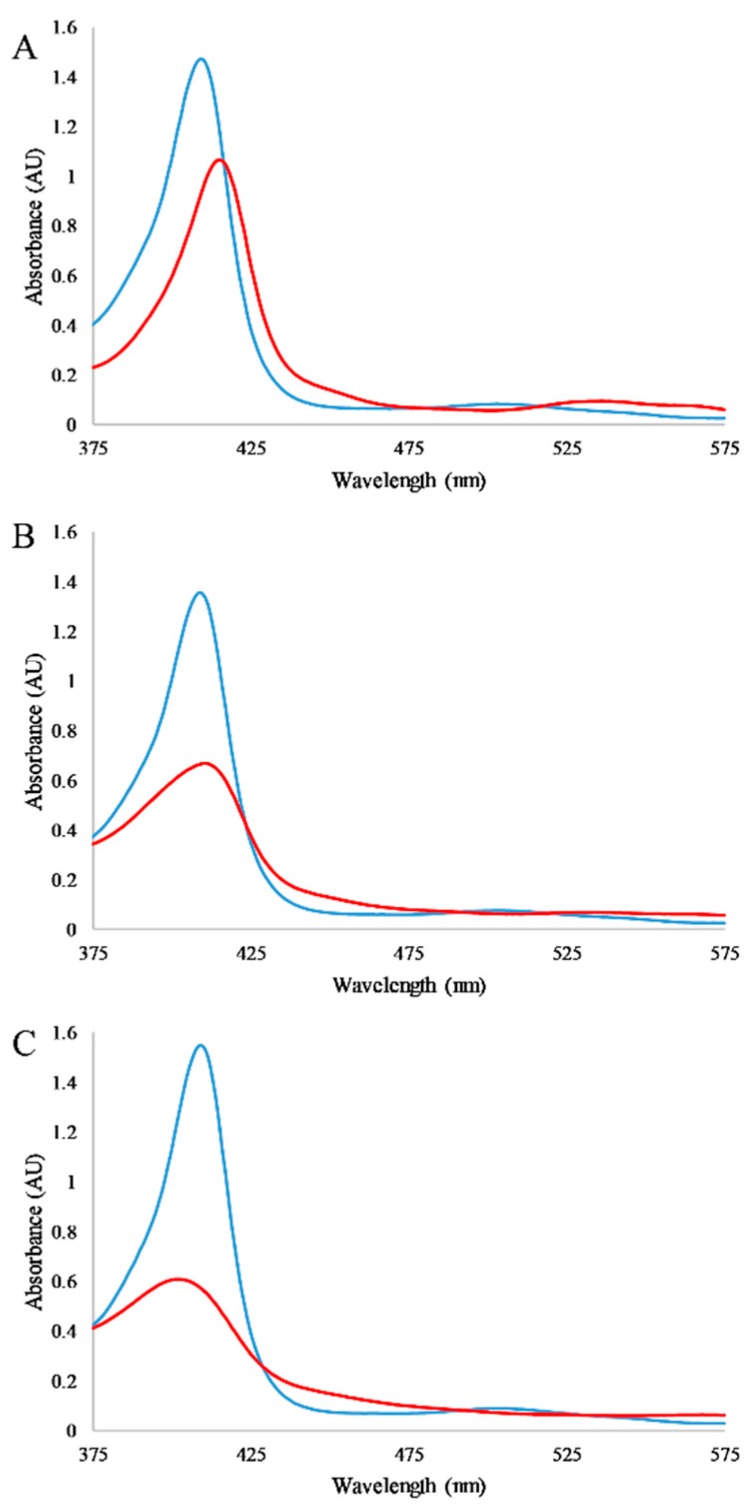
Absorbance spectra of myoglobin and detergents. Spectra of 0.2 mg/mL myoglobin in the absence (blue lines) and presence (red lines) of detergents: (**A**) 1 mM SDS, (**B**) 2 mM EBB, (**C**) 2.5 mM TTAB. All spectra are representative examples after 30 min of incubation with the detergent; samples were in 2 mM phosphate buffer at pH 7.

The absorbance of the myoglobin heme group in the visible region and the sensitivity of this absorption to local protein. Exploiting this spectroscopic sensitivity of myoglobin, absorbance spectra were recorded for the protein in the presence and absence of detergents and in the presence and absence of the RTILs alone (Figure 2). In the case of detergents, as expected the heme absorbance peak at 409 nm decreases upon exposure to detergents; however, it is notable that the spectra in the presence of detergents differ in both the spectral absorption, peak shape, and peak position. These spectra may indicate differences in the final denatured conformation of the protein or the extent of the heme dissociation. Control experiments in which CD spectra of myoglobin were collected in the presence of detergent, but in the absence of RTIL, show that the detergent alone does not cause significant denaturation of the protein structure (Supplementary Figure S2). Thus, the intensity at 409 nm is a measure of the dissociation of heme from myoglobin as a response to detergent and/or RTIL.

**Figure 3:**
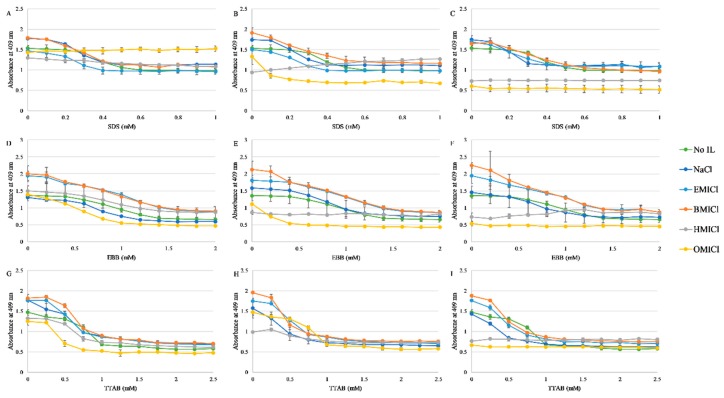
Dissociation of heme from myoglobin by absorbance spectroscopy. Color coding for each RTIL or control is indicated in the figure. Panels (**A**–**C**) are with SDS, (**D**–**F**) are with EBB, and (**G–I**) are with TTAB. Panels (**A**), (**D**), and (**G**) (left column) are supplemented with 50 mM of RTIL; panels (**B**), (**E**), and (**H**) are supplemented with 150 mM RTIL; panels (**C**), (**F**), and (**I**) are supplemented with 300 mM RTIL. Data represent the average of at least three replicates, error bars represent the standard deviation, and lines are not fits but are only to guide the eye.

Detergent mediated dissociation of the heme group was then monitored for each detergent in the presence of 50, 150, or 300 mM RTIL, or NaCl as a control. The results of these experiments are shown in Figure 3 with representative spectra of myoglobin in with each RTIL shown in Supplementary Figure S3. Notably, HMICl and OMICl appear to significantly destabilize the myoglobin at both 150 and 300 mM concentrations such that the heme absorbance is significantly reduced prior to any addition of detergent, resulting in an effectively flat titration profile. In the remainder of cases, the protein exhibited a traditional sigmoidal dissociation profile as a function of detergent concentration for both TTAB and SDS. The dissociation induced by EBB was a broader transition, similar to what was previously observed.

### 3.3. Fluorescence Spectroscopy

**Figure 4:**
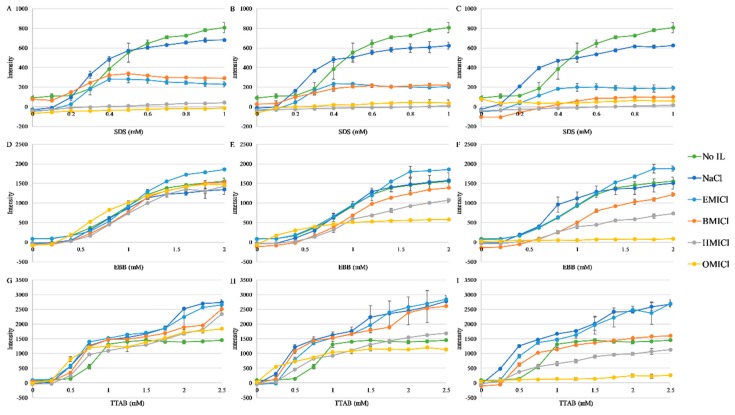
Dissociation of heme from myoglobin by fluorescence dequenching. Data are the emission intensity of the sample with λex = 280nm, λem = 340nm. Color coding for each IL or control is indicated in the figure. Panels (**A**–**C**) are with SDS, (**D**–**F**) are with EBB, and (**G–I**) are with TTAB. Panels (**A**), (**D**), and (**G**) (left column) are supplemented with 50 mM of RTIL; panels (**B**), (**E**), and (**H**) are supplemented with 150 mM RTIL; panels (**C**), (**F**), and (**I**) are supplemented with 300 mM RTIL. Data represent the average of at least three replicates, error bars represent the standard deviation, and lines are not fits but are only to guide the eye.

The myoglobin sequence has two Trp residues that, when in the fully-folded holo-form, are buried at the protein interior and are close to the heme group. This proximity results in heavy atom-based quenching of the Trp fluorescence emission, which is relieved by the increased distance between Trp and heme upon heme dissociation and/or myoglobin unfolding (Figure 1). Based on the most recently-released crystal structure, PDBID:5ZZE, the Trp residues are ~19 Å from the heme based on heme-iron to indole nitrogen measurements, well within the typical distances for heavy atom quenching. Thus, increases in myoglobin Trp fluorescence were also used as a means of monitoring heme dissociation or protein unfolding. Experiments parallel to the absorbance experiments in Figure 3 were carried out and the results are shown in Figure 4. Generally, the majority of conditions exhibit a traditional sigmoidal dissociation/denaturation profile as a function of detergent concentration. However, in this case, the more sensitive fluorescence assay indicates that some conditions that exhibited a flat dissociation profile in the absorbance assay (e.g., 150 mM HMICl in EBB) do exhibit a sigmoidal transition in fluorescence (Figure 4E). While these are more sensitive, quantitative analysis of dissociation from these values cannot be accurately calculated due to potentially differential contributions of fluorescence quenching by the RTILs. Overall, the trends are similar to those seen in Figure 3 with increased sensitivity to heme dissociation.

### 3.4. Circular Dichroism Spectroscopy

**Figure 5:**
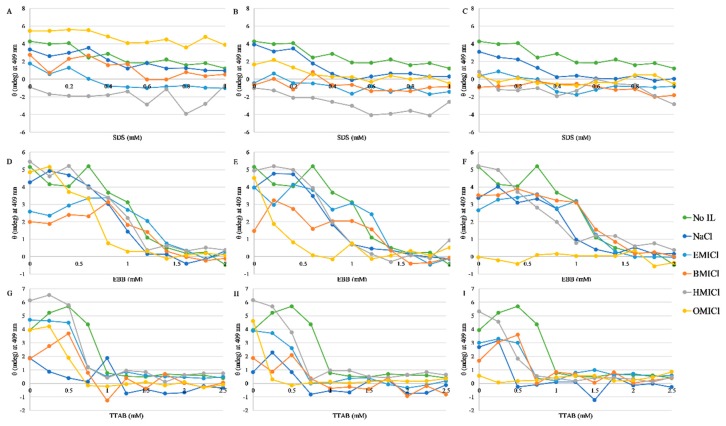
Dissociation of heme from myoglobin by circular dichroism spectroscopy. Data were collected at 409 nm. Color coding for each IL or control is indicated in the figure. Panels (**A–C**) are with SDS, (**D**–**F**) are with EBB, and (**G–I**) are with TTAB. Panels (**A**), (**D**), and (**G**) (left column) are supplemented with 50 mM of RTIL; panels (**B**), (**E**), and (**H**) are supplemented with 150 mM RTIL; panels (**C**), (**F**), and (**I**) are supplemented with 300 mM RTIL. Data represent the average of 16 replicate scans of a single representative sample and lines are not fits but are only to guide the eye.

Due to the high absorptivity of the imidazolium group in the low-UV region, traditional CD spectroscopy monitoring protein structure (190–250 nm range) cannot be faithfully performed in the presence of these RTILs. However, the heme group also exhibits a CD-active band near 409 nm when bound in the folded myoglobin structure. This band is also sensitive to local protein structure, and was; thus, used to confirm the heme dissociation results from the absorbance and fluorescence results. The CD values as a function of detergent in each of the ionic liquids is shown in Figure 5. Representative spectra are shown in Supplementary Figure S4.

### 3.5. Thermodynamic Analysis of Heme Dissociation

**Figure 6:**
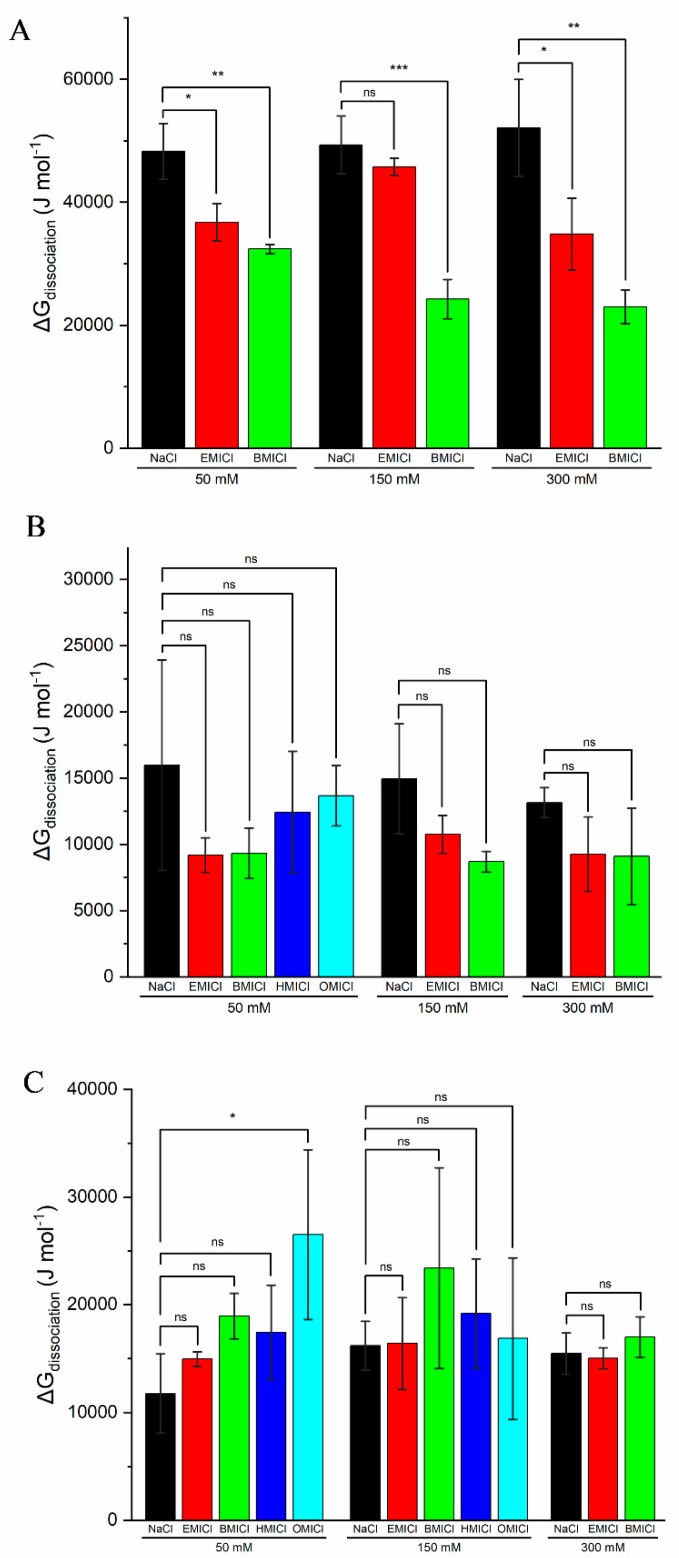
Thermodynamic values and statistical analysis for ΔG_dissociation_ for heme by (**A**) SDS, (**B**) EBB, or (**C**) TTAB. ΔG_dissociation_ values are calculated as the average of individual replicates with error bars representing standard deviation of the values calculated from each trial. Statistical analysis was performed by SAS Institute’s JMP Pro 13 (Cary, NC, USA) with ns = no significant difference, * *P* ≤ 0.05, ** *P* ≤ 0.01, and *** *P* ≤ 0.001.

Based on the absorbance data in Figure 3, the Gibbs free energy of dissociation was calculated for each of the RTILs and detergents in this study where clear dissociation curves were evident using Equation (1). This calculation was performed as previously reported and representative samples of the fitting is shown in Supplementary Figure S5 [12]. The ΔG_dissociation_ values for each detergent and RTIL concentration tested are shown in Figure 6. Statistical analysis was performed to compare ΔG_dissociation_ in the presence of NaCl to the equivalent concentrations of RTIL in an effort to eliminate the contribution of solution ionic strength to any changes in ΔG_dissociation_. Interestingly, there was no statistical difference found in the ΔG_dissociation_ for dissociation induced by EBB with any RTIL, or by TTAB with any IL except 50 mM OMICl. Alternatively, statistical differences were found with SDS and all concentrations of BMICl and EMICl except 150 mM EMICl. Notably, HMICl and OMICl were omitted from numerous calculations and analyses because dissociation of heme was complete or near complete in the absence of detergent.

## 4. Discussion

Understanding and predicting the interaction of RTILs with biomolecules is a critical step in the development and application of RTILs to a number of fields including pharmaceuticals and medical devices [9]. This understanding is inherently complex, as there are a wide variety of molecular structures that compose RTILs and an even wider complexity of biomolecules. 

Myoglobin is a well characterized protein that has often been used for protein denaturation studies due to the favorable spectroscopic properties. The structure of myoglobin consists exclusively of α-helices, making structural studies with CD spectroscopy very straightforward, thus allowing for a strong understanding of the inherent protein stability to be established. The majority of detergent denaturation studies on myoglobin have focused on the commonly used surfactant SDS. In particular, Otzen and coworkers have shown in a number of studies that the SDS-induced myoglobin unfolding pathway is a multistep process with numerous intermediates [27,28]. In a capillary electrophoresis assay, myoglobin and other proteins were shown to differentially respond to SDS with respect to denaturation state, pathway, and kinetics [29]. Taken together, these findings are consistent with our results in that 2 mM SDS does not completely unfold the myoglobin but does induce loss of the heme group.

Beyond SDS, very little has been reported in the literature about detergent-mediated denaturation of myoglobin. Again, Otzen and coworkers characterized the interaction of the anionic rhamnolipid surfactants with myoglobin and found differences in protein affinity and denaturation kinetics [30]. Regarding zwitterionic detergents, our previous work showed that EBB does not denature the structure of myoglobin up to 8 mM EBB, well beyond the concentrations required to cause heme dissociation [12]. There are several reports in the literature on zwitterionic detergent interactions with myoglobin; however, these are focused on the effects the detergent have on mass spectrometry detection and signal suppression [31,32]. Cationic detergents have wider application in life sciences than zwitterionic detergents; however, the application of these molecules to protein denaturation is still somewhat limited. Additionally, even among the reports which use cationic detergents as protein denaturants, there is no single detergent that is a standard denaturant as compared to the widespread utility of SDS. The most commonly used cationic detergent is cetyltrimethylammonium chloride (CTAC) and the closely related cetyltrimethylammonium bromide (CTAB), which differ only in the counterion. Several groups have shown a biphasic response of myoglobin to CTAC, a slow and minor alteration of structure below the CMC, and a larger change in protein structure when detergent concentrations surpass the CMC [27,33]. More closely related to the work presented in this paper, Mandal and Ghosh demonstrated that the additive curcumin partially inhibited the denaturation of myoglobin by CTAB [34]. Notably, all of these studies employed CTAB/CTAC which has a 16C alkyl chain, while the TTAB used in this work has a 14C alkyl chain. Although this is a relatively small change, the two detergents show ~4-fold difference in CMC, which clearly impacts the denaturation of myoglobin [35]. It is unclear, and an area of future study, how these small differences in detergent structure may affect the denaturation profile of myoglobin and other proteins with RTILs.

As previous reports have shown, many RTILs do not induce complete protein structural denaturation on their own, but instead induce nuanced changes that affect protein stability, enzymatic activity, or ligand binding [1,5,10,12]. Similarly, our results show that myoglobin is not dramatically affected by the presence of any of the RTILs tested at 50 mM, but the absorbance spectra are noticeably shifted when exposed to 150 mM OMICl and 300 mM HMICl or OMICl (Supplementary Figure S3). Thus, in order to tease out the effects of various RTILs on protein properties, they have often been mixed with traditional chaotropes to enhance or amplify the activity to gain a better understanding of mechanism. There are numerous proposed models for how RTILs destabilize proteins, including through disruption of H-bonds, ionic interactions, or the disruption of hydrophobic contacts at the protein interior [36,37]. The latter model has been proposed for molecules such as OMICl which has a long alkyl chain that can behave in a detergent-like manner [38,39,40]. Several groups have reported the inherent propensity to micellize of some ionic liquids, a property that is directly linked to alkyl chain length [24,41]. Thus, combining these detergent-like RTILs with standard detergents could enhance the protein denaturation if both molecules are acting via the same mechanism. However, this is not supported by the data presented herein. Overall, RTILs EMICl and BMICl showed no significant effect on dissociation by EBB and TTAB, but showed varying degrees of enhanced dissociation by SDS (Figure 6). In most cases, the HMICl and OMICl RTILs induced enough heme dissociation in the absence of detergent that reliable thermodynamic calculations and statistical analysis could not be performed.

The results and analysis above highlight two important findings regarding the RTIL interactions with myoglobin and detergent. The first important result is the inherent ability of the longer chain RTILs to cause dissociation of heme. While this result is not inherently surprising considering the well documented micellization propensity of these molecules, it highlights implications for the application of RTILs with biomolecules. Future studies on long chain RTILs should be performed keeping alkyl chain-mediated differential activity in mind despite identical charge-bearing moieties, in this case the imidazolium. Additionally, any enhancement of protein denaturation or ligand dissociation, and specifically heme dissociation, by HMICl or OMICl must be performed at significantly lower concentration regimes than presented herein. The long-chain RTIL concentration dependence on enhancement of detergent-mediated dissociation of heme is an interesting area for future study. These findings then support a model of RTIL association to the surface of the protein, as presented in a number of MD simulation studies [42,43,44,45]. Specifically, several MD simulation studies have shown that the RTILs BMICl, BMIPF_6_, and BMINO_3_ exhibit a similar behavior in which the imidazolium cation associates with the surface of the protein [44,45]. In the context of this study, imidazolium partitioning to the protein surface could then result in the intercalation of the alkyl chain into the protein interior. Thus, longer alkyl chains can partition deeper into the protein hydrophobic core, resulting in greater protein destabilization and heme dissociation. This mechanism is similar to that proposed by Jha et al. for hemoglobin [22]. This model is similar to a traditional detergent model, but appears to be more strongly influenced by the imidazolium association to the surface rather than the hydrophobic partitioning of the alkyl chain.

Focusing on the short alkyl chain RTILs EMICl and BMICl, it is clear that not all detergents interact with RTILs in the same way. Both EBB (zwitterionic) and TTAB (cationic) exhibited no significant impact from the short chain RTILs, while dissociation by SDS (anionic) was affected by all RTILs examined. This implies that there is either an inhibition of activity by EBB and TTAB or an enhancement of dissociation by SDS. The enhancement model can be supported by favorable interactions between the cationic component of the RTIL, which is the active species in these studies, and the anionic headgroup of the SDS detergent molecules. Several reports have shown favorable interactions between imidazolium-based RTILs and SDS, although the most pronounced effects have been with longer alkyl chains (C6 and C12) [46,47,48,49]. These electrostatic interactions may aid in recruitment or binding of the oppositely charged species to the myoglobin protein, increasing the local concentration and/or partitioning to the hydrophobic heme pocket. Alternatively, a bound complex between SDS and RTIL molecules may result in a more bulky species to disrupt local packing of the myoglobin near the heme pocket. Notably, the CD spectra of RTILs with SDS display a significant spectral shape change after addition of SDS compared to EBB and TTAB (Supplementary Figure S4), indicating this interaction is indeed resulting in a different local environment around the heme. Sequential binding experiments using physical methods are necessary to elucidate the more complex details of this process

## 5. Conclusions

The results presented herein highlight the significant impact of relatively small changes in alkyl chain length (from C4 to C6), and underscores the need for careful experimentation and controls during experiments with RTILs and biomolecules. These results support the hypothesis that imidazolium ion partitions to the surface of the protein, and the alkyl chain partitions into the hydrophobic core of the protein destabilizing the heme. More specifically, the enhancement of heme dissociation by the anionic detergent SDS indicates that solution-based ionic interactions may be used to modulate RTIL interactions with biomolecules. Further experiments must be carried out to elucidate whether this enhanced dissociation is specific to SDS, more general for anionic detergents, or related to the molecular structure of the SDS headgroup. Finally, taken together, the results show the rich diversity of activities that very similar RTILs have with biomolecules, and open the door to new potential applications of long chain RTILs as denaturants or denaturation additives for very stable proteins or protein complexes.

## Supplementary Materials

The following are available online at https://www.mdpi.com/2218-273X/9/7/264/s1, Supplementary Figure S1—CMC analysis. Supplementary Figure S2—CD spectra of myoglobin in detergent. Supplementary Figure S3—Representative absorbance spectra of myoglobin in the presence of differing concentrations of RTILs. Supplementary Figure S4—Representative CD spectra of myoglobin in the presence of 50 mM EMICl and differing concentrations of detergent. Supplementary Figure S5—Sample data fitting for ΔG_dissociation_ calculations. Supplementary Table S1—Sample compositions tested.
