# Supplementary material for: Effects of Ionic Liquid Alkyl Chain Length on Denaturation of Myoglobin by Anionic, Cationic, and Zwitterionic Detergents
Source: Biomolecules. 2019 Jul 8;9(7):264. doi: 10.3390/biom9070264 (PMC6681400; doi:10.3390/biom9070264)
Supplement: Supplementary file 1 [file biomolecules-09-00264-s001.pdf]

biomolecules

MDPI

*Supplementary Information*

# Effects of Ionic Liquid Alkyl Chain Length on Denaturation of Myoglobin by Anionic, Cationic, and Zwitterionic Detergents

**Joshua Y. Lee [1,2], Katherine M. Selfridge [1], Eric M. Kohn [1,2], Timothy D. Vaden [1] and Gregory A. Caputo [1,3,*]**

[1] Department of Chemistry and Biochemistry, Rowan University, Glassboro NJ 08028 USA; leej8@students.rowan.edu (J.Y.L.); selfridgk9@students.rowan.edu (K.M.S.); kohne2@students.rowan.edu (E.M.K.); vadent@rowan.edu (T.D.V.)

[2] Bantivoglio Honors College, Rowan University, Glassboro NJ 08028 USA

[3] Department of Molecular and Cellular Biosciences, Rowan University, Glassboro NJ 08028 USA

* Correspondence: caputo@rowan.edu

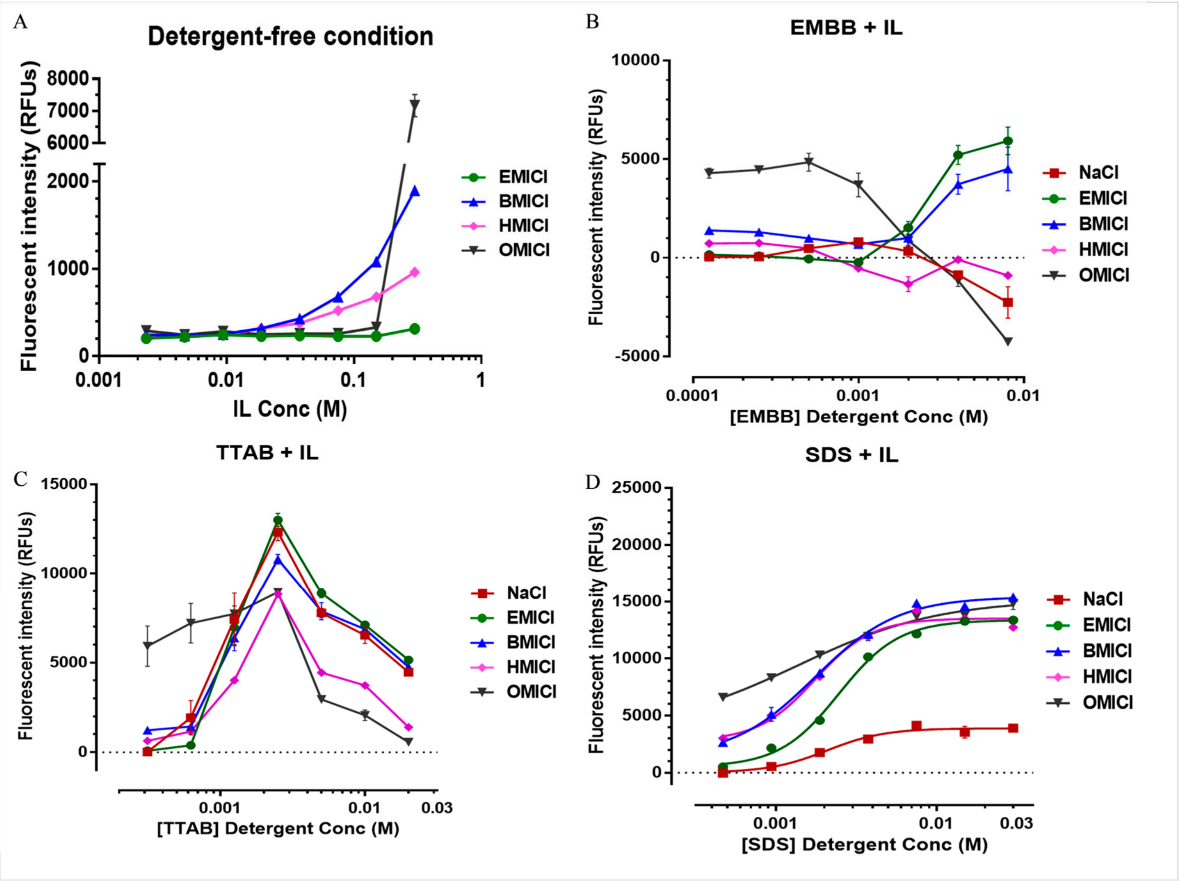


**Supplementary Figure S1. CMC analysis.** CMC determination based on DPH fluorescence for each RTIL (**A**), and detergents (**B**) EBB, (**C**) TTAB, and (**D**) SDS are shown. In each panel colors are : EMICl – green, BMICl – blue, HMICl – pink, OMICl – black, and NaCl – red. Note the log-scale for the X-axis. Sample data were corrected for background fluorescence in samples lacking DPH. Data were analyzed using the Prism software package. Error bars represent the standard deviation of three samples.

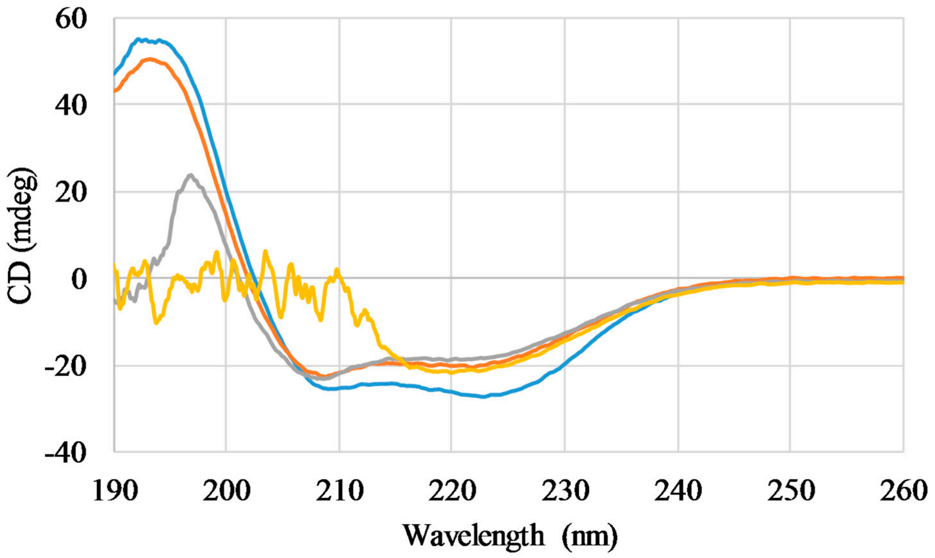


**Supplementary Figure S2.** CD spectra of myoglobin in detergent**.** CD spectra were collected in 2 mM phosphate buffer (blue) or 2 mM phosphate supplemented with the following detergents: 1.0 mM SDS (orange), 2.0 mM EBB (gray), or 2.5 mM TTAB (yellow). All samples were the average of 16–64 scans and were background corrected by subtracting spectra lacking protein.

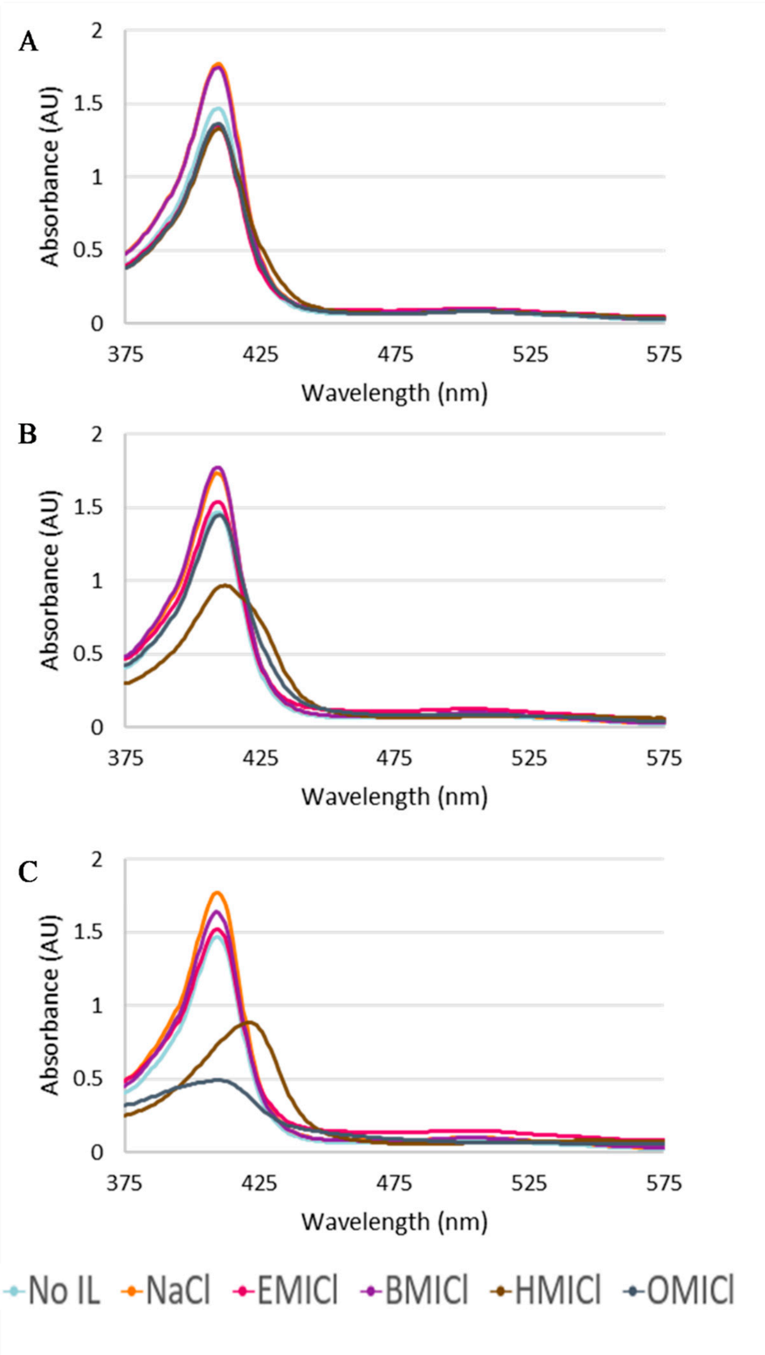


**Supplementary Figure S3.** Representative absorbance spectra of myoglobin in the presence of differing concentrations of RTILs. (**A**) 50 mM, (**B**) 150 mM, (**C**) 300 mM. Color coding of spectra for all panels is indicated at the bottom of the Figure.

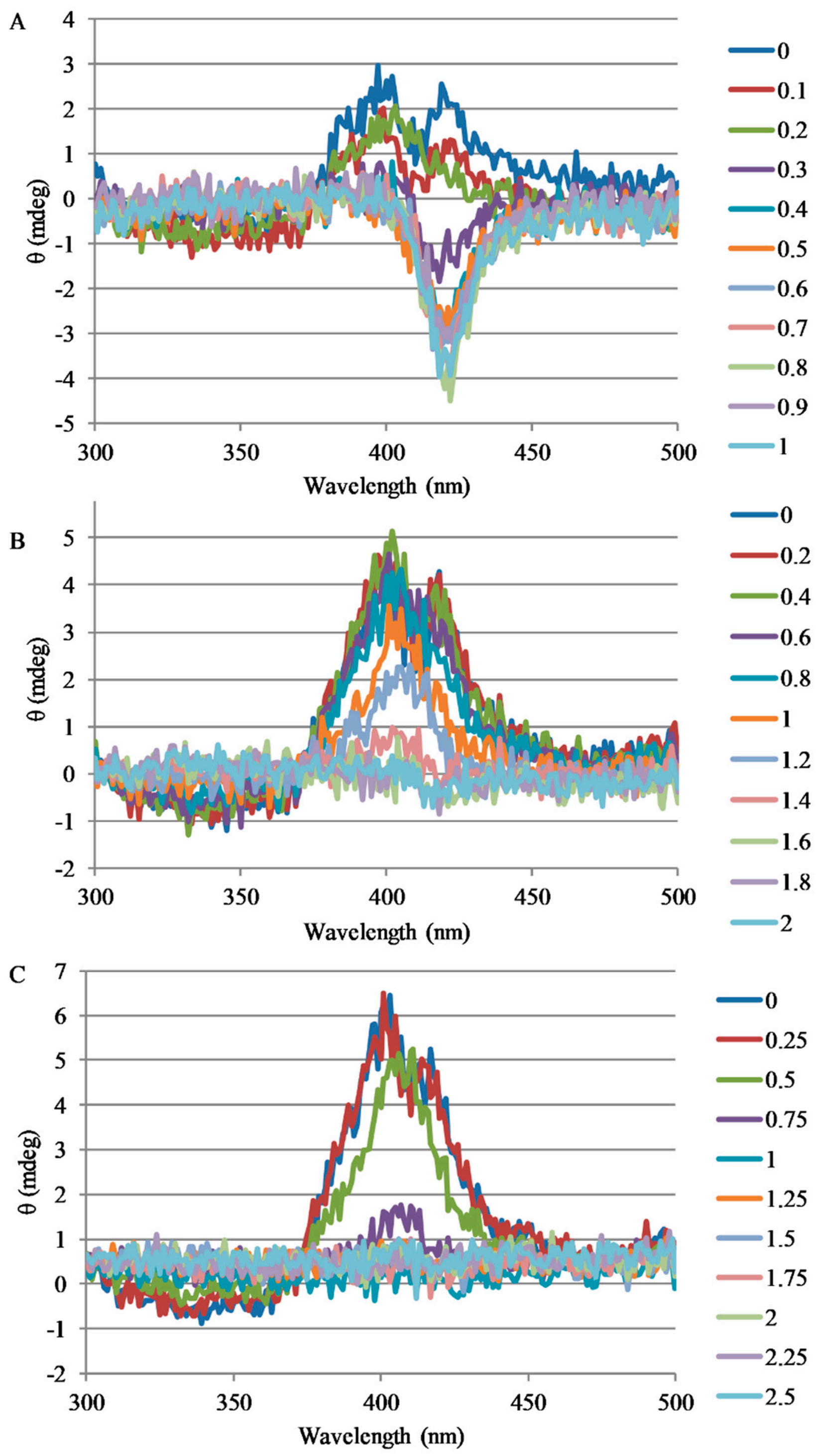


**Supplementary Figure S4.** Representative CD spectra of myoglobin in the presence of 50 mM EMICl and differing concentrations of detergent. (**A**) SDS, (**B**) EBB, (**C**) TTAB. Color coding of spectra for all panels is indicated in each panel of the Figure.

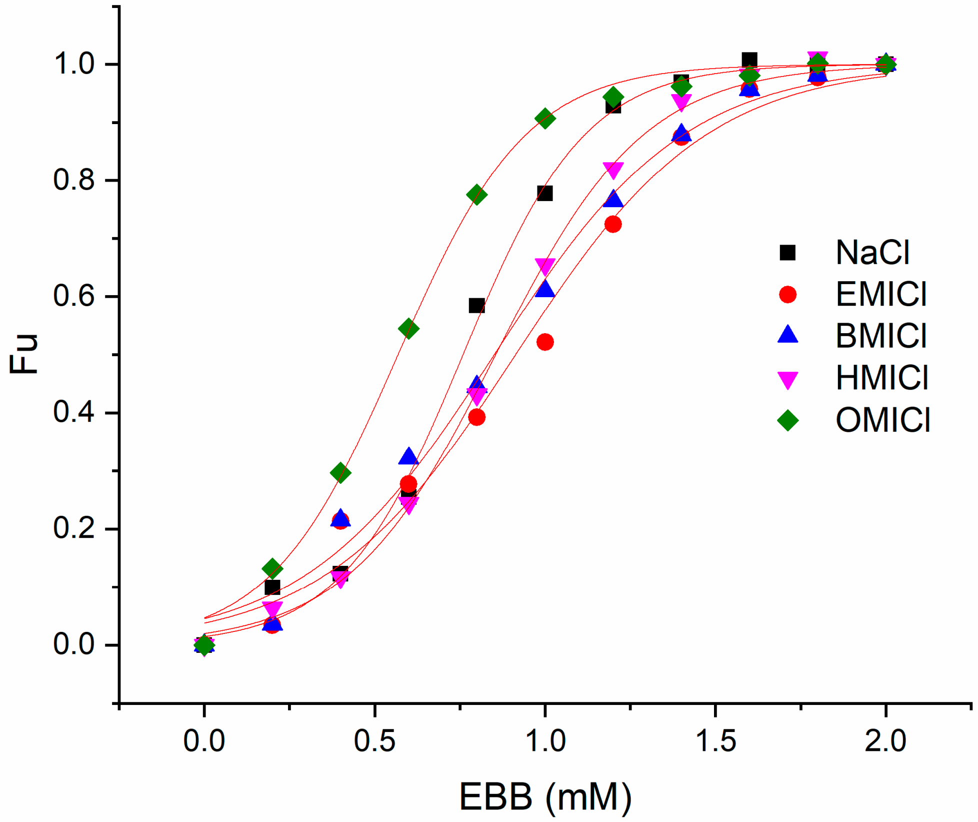


**Supplementary Figure S5.** Sample data fitting for $\Delta G_{dissociation}$ calculations. Data represent individual data sets of myoglobin with 50 mM RTIL denatured with varying concentrations of EBB. Fu represents fraction unbound, that is, the fraction of heme that has been dissociated.

**Supplementary Table S1.** Sample Compositions.

| Detergent (mM) | | Ionic Liquid or NaCl control (mM) | | | |
|---|---|---|---|---|---|
| | | 0 | 50 | 150 | 300 |
| SDS | 0.00 | + | + | + | + |
| | 0.10 | + | + | + | + |
| | 0.20 | + | + | + | + |
| | 0.30 | + | + | + | + |
| | 0.40 | + | + | + | + |
| | 0.50 | + | + | + | + |
| | 0.60 | + | + | + | + |
| | 0.70 | + | + | + | + |
| | 0.80 | + | + | + | + |
| | 0.90 | + | + | + | + |
| | 1.00 | + | + | + | + |
| EBB | 0.00 | + | + | + | + |
| | 0.20 | + | + | + | + |
| | 0.40 | + | + | + | + |
| | 0.60 | + | + | + | + |
| | 0.80 | + | + | + | + |
| | 1.00 | + | + | + | + |
| | 1.20 | + | + | + | + |
| | 1.40 | + | + | + | + |
| | 1.60 | + | + | + | + |
| | 1.80 | + | + | + | + |
| | 2.00 | + | + | + | + |
| TTAB | 0.00 | + | + | + | + |
| | 0.25 | + | + | + | + |
| | 0.50 | + | + | + | + |
| | 0.75 | + | + | + | + |
| | 1.00 | + | + | + | + |
| | 1.25 | + | + | + | + |
| | 1.50 | + | + | + | + |
| | 1.75 | + | + | + | + |
| | 2.00 | + | + | + | + |
| | 2.25 | + | + | + | + |
| | 2.50 | + | + | + | + |

© 2019 by the authors. Licensee MDPI, Basel, Switzerland. This article is an open access article distributed under the terms and conditions of the Creative Commons Attribution (CC BY) license (http://creativecommons.org/licenses/by/4.0/).
